# The Neuropsychiatric Inventory Questionnaire and Mini-Mental State Examination in low- and middle-income countries: correlational insights from a Vietnamese memory clinic sample

**DOI:** 10.1590/1980-5764-DN-2025-0326

**Published:** 2025-10-26

**Authors:** Quynh-Nga Pham, Vinh-Khang Nguyen, Cong-Thang Tran

**Affiliations:** 1 University of Medicine and Pharmacy at Ho Chi Minh City, Vietnam.; 2 University Medical Center HCMC, Department of Neurology, Ho Chi Minh City, Vietnam.

**Keywords:** Dementia, Behavioral Symptoms, Mental Disorders, Cognitive Dysfunction, Mental Status and Dementia Tests, Alzheimer Disease, Demência, Sintomas Comportamentais, Transtornos Mentais, Disfunção Cognitiva, Testes de Estado Mental e Demência, Doença de Alzheimer

## Abstract

In low- and middle-income countries, dementia awareness remains limited; behavioral and psychological symptoms of dementia (BPSD) are often misunderstood and underutilized in early screening.

**Objective:**

To examine the correlation between cognitive and behavioral symptoms using the Mini-Mental State Examination (MMSE) and the Neuropsychiatric Inventory Questionnaire (NPI-Q) in a Vietnamese clinical sample.

**Methods:**

A total of 98 participants aged 40 and above with memory complaints were recruited from the outpatient dementia clinic at University Medical Center, Ho Chi Minh City. All participants underwent neurological and physical examinations, cognitive screening using the Vietnamese version of the MMSE, and behavioral assessment using the Vietnamese NPI-Q completed by caregivers. Brain imaging via computed tomography (CT) or magnetic resonance imaging (MRI) was also performed. Participants were categorized based on MMSE scores, and correlations between MMSE and NPI-Q scores were analyzed.

**Results:**

BPSD was present in 95.9% of participants. Anxiety was most frequent in individuals with subjective memory complaints, while psychotic symptoms and apathy were more common in those with severe cognitive impairment. A moderate negative correlation was found between MMSE and NPI-Q scores.

**Conclusion:**

The combined use of MMSE and NPI-Q, supported by brain imaging, may provide a practical approach for the initial screening of individuals with memory complaints, particularly in low- and middle-income countries (LMICs) where access to specialist resources is limited.

## RESUMO.

Em países de renda baixa e média, a conscientização sobre a demência ainda é limitada, e os sintomas comportamentais e psicológicos da demência (SCPD) frequentemente são mal compreendidos e subutilizados nas triagens iniciais.

**Objetivo:**

Examinar a correlação entre sintomas cognitivos e comportamentais utilizando o Mini Exame do Estado Mental (MEEM) e o Questionário de Inventário Neuropsiquiátrico (Neuropsychiatric Inventory Questionnaire – NPI-Q) em uma amostra clínica vietnamita.

**Métodos:**

Um total de 98 participantes com 40 anos ou mais, com queixas de memória, foi recrutado na clínica ambulatorial de demência do Centro Médico Universitário, na Cidade de Ho Chi Minh. Todos os participantes foram submetidos a exames neurológicos e físicos, triagem cognitiva com a versão vietnamita do MEEM e avaliação comportamental por meio do questionário NPI-Q vietnamita, preenchido pelos cuidadores. Também foram realizados exames de imagem cerebral por tomografia computadorizada (TC) ou ressonância magnética (RM). Os participantes foram categorizados com base nos escores do MEEM, e as correlações entre os escores do MEEM e do NPI-Q foram analisadas.

**Resultados:**

Os SCPD estavam presentes em 95,9% dos participantes. Ansiedade foi o sintoma mais frequente entre indivíduos com queixas subjetivas de memória, enquanto sintomas psicóticos e apatia foram mais comuns naqueles com comprometimento cognitivo grave. Foi encontrada uma correlação negativa moderada entre os escores do MEEM e do NPI-Q.

**Conclusão:**

O uso combinado do MEEM e do NPI-Q, apoiado por exames de imagem cerebral, pode representar uma abordagem prática para a triagem inicial de indivíduos com queixas de memória, especialmente em países de baixa e média renda, onde o acesso a recursos especializados é limitado.

## INTRODUCTION

 According to the World Health Organization, Vietnam is one of the fastest-aging countries in the world. The number of people living with dementia in Vietnam is projected to rise from 660,000 in 2015 to 1.2 million by 2030 and 2.4 million by 2050^1^. Memory complaints are among the earliest symptoms of Alzheimer disease (AD) and dementia in middle-aged populations and may serve as predictors of cognitive impairment^2^. In addition to cognitive decline, behavioral and psychological symptoms of dementia (BPSD) can occur at any stage of cognitive impairment, including the early stages^3^. BPSD are prevalent, with most dementia patients experiencing such symptoms at some point during the course of the illness. These symptoms are diverse and unpredictable, and may vary significantly in frequency and intensity within the same individual. The impact of BPSD is substantial, contributing to significant distress for patients, considerable strain on caregivers, and increased societal costs. Behavioral changes such as agitation, anxiety, or psychotic symptoms in dementia patients are particularly stressful for caregivers^4^. 

 In Vietnam, social and medical support specifically for dementia patients remains inadequate, and the lack of emphasis on early dementia diagnosis presents challenges to providing continuous care^5^. Due to cultural factors, the relationship between BPSD and cognitive performance has been rarely reported in early studies on dementia in Vietnam. BPSD is not systematically assessed in clinical settings in the country. Cognitive and behavioral symptoms of dementia remain significantly under-recognized, underreported, inadequately treated, and poorly managed, particularly in low- and middle-income countries (LMICs)^6^. Numerous studies have demonstrated a correlation between cognitive assessment and behavioral symptoms; however, most were conducted in developed countries with earlier detection and treatment of dementia or in different populations^7-9^. The Mini-Mental State Examination (MMSE) is a widely used cognitive screening tool in Vietnam, while the Neuropsychiatric Inventory Questionnaire (NPI-Q) is employed to assess BPSD. Nonetheless, the combined use of these tools for early dementia screening has not been widely investigated in the Vietnamese context. 

 This study aimed to explore the relationship between MMSE and NPI-Q scores in individuals presenting with memory complaints and to evaluate the potential utility of combining both instruments for early cognitive and behavioral assessment. 

## METHODS

### Translation and cross-cultural adaptation of the NPI-Q

 The NPI-Q used in this study was translated into Vietnamese with permission from Dr. Jeffrey Cummings^10^. Forward translation into Vietnamese was performed by two independent bilingual Vietnamese-English translators. These translations were compared and discussed, resulting in a draft version. Two additional bilingual translators conducted a back-translation, and the resulting versions were reviewed by a panel of three neurologists in comparison with the original English NPI-Q. The content validity of the draft translation was assessed by two neurologists who had not participated in the initial translation process. A pre-final version of the Vietnamese translation was administered in a pilot study involving ten caregivers of dementia patients to assess the clarity and comprehensibility of the test items. None of the caregivers reported difficulties in understanding the scoring scale, leading to the finalization of the Vietnamese NPI-Q. 

### Participants

 Ninety-eight individuals aged 40 and above with subjective memory complaints were consecutively recruited from the dementia outpatient clinic at the University Medical Center, Ho Chi Minh City. Inclusion criteria required participants to be 40 years old or older. Individuals with severe hearing or visual deficits, major psychiatric disorders (*e.g.*, major depression, obsessive-compulsive disorder – OCD, bipolar disorder, schizophrenia, autism, and delirium), or those receiving medical treatment for dementia or BPSD were excluded. Additional exclusion criteria included medical conditions such as stroke, multiple sclerosis, Parkinson’s disease, traumatic brain injury, epilepsy, and encephalitis. 

 Each participant underwent a structured clinical evaluation, including physical and neurological examinations, conducted by board-certified neurologists experienced in cognitive disorders. Brain imaging (computed tomography – CT or magnetic resonance imaging – MRI) was performed for all participants. Cognitive assessment was conducted using the Vietnamese version of MMSE. NPI-Q was completed by caregivers who had lived with the patients for at least 3 months. 

 Although MMSE is not a diagnostic tool for dementia, it was used in this study to stratify cognitive severity for correlation analysis with NPI-Q scores. MMSE score ranges were: 0–13 (severe), 14–19 (moderate), 20–23 (mild), and ≥24 (subjective complaints). This grouping was intended solely for analytical purposes, not diagnostic categorization. 

 NPI-Q provides information on the behavioral symptoms of dementia. It assesses 12 domains including delusions, hallucinations, agitation/aggression, depression/dysphoria, anxiety, euphoria, apathy/indifference, disinhibition, irritability/lability, aberrant motor activity, nighttime behavioral disturbances, and appetite/eating abnormalities. NPI-Q is designed to be a self-administered questionnaire completed by caregivers, who were either family members or hired caregivers residing with the patients for at least the past three months. Most caregivers completed the NPI-Q in five minutes or less. A neurologist interviewed all caregivers to clarify any uncertain responses. Each domain was assessed using a screening question that determined the presence (0: no; 1: yes) and severity (1/3: mild; 2/3: moderate; 3/3: severe) of symptoms during the previous month. The total NPI-Q severity score is the sum of the individual symptom scores, ranging from 0 to 36. Caregiver distress related to each neuropsychiatric symptom was rated on a six-point Likert scale, ranging from 0 ("not distressing at all") to 5 ("extremely distressing"). The total distress score is the sum of these ratings, ranging from 0 to 60. 

### Statistical analysis

 In this study, various statistical methods were applied to assess the relationships between cognitive impairment, neuropsychiatric symptoms, and caregiver distress. Descriptive statistics were used to summarize demographic information and key variables such as age, MMSE scores, and NPI-Q scores, presented as medians with interquartile ranges, means with standard deviations, and percentages, as appropriate. The χ^2^ test was employed to compare the proportions of neuropsychiatric symptoms across different levels of cognitive impairment, aiming to evaluate their association with dementia severity. The Kruskal -Wallis test was conducted to assess differences in NPI-Q scores and caregiver distress among groups with mild, moderate, and severe cognitive impairment, followed by post-hoc analysis to identify specific group differences. Spearman’s rank correlation was conducted to examine the relationship between MMSE and NPI-Q scores, exploring the link between cognitive decline and neuropsychiatric symptoms. 

## RESULTS

### Patient characteristics

 The mean age was 65.52±10.35 years. Participants aged over 80 accounted for 8.2%, while those aged 60–80 comprised 69.4%. Females constituted 60.7% of the sample, and males 39.3%. Participants with low educational levels (below the 5th grade) made up 35.7% of the sample. The study population was evenly divided into four groups based on cognitive impairment severity, as determined by MMSE scores. 

Table 1 presents the number of patients who met the inclusion and exclusion criteria for dementia stages, classified according to MMSE scores. Table 2 displays the prevalence of individual NPI-Q domain scores across patients with varying cognitive impairment severity based on MMSE score. The overall prevalence of any behavioral and psychological symptoms across the entire patient group was 95.9%. 

**Table 1:** Patient characteristics.

Characteristics		Number	Percentage %
Age group (years old)	<59	22	22.4
	60–80	68	69.4
	>80	8	8.2
Gender	Male	30	39.3
	Female	68	60.7
Educational level	No education	1	1
	Grade 1–5	34	34.7
	Grade 6–9	23	23.8
	Grade 10–12	31	31.6
	University level	8	8.2
	Posgraduate level	1	1
Occupation	Intellectual work	25	25.5
	Other types of work	73	74.5
Groups classified by MMSE score	Subjective memory complaint (MMSE≥24)	28	28.57
	Mild dementia	25	25.51
	Moderate dementia	27	27.55
	Severe dementia	18	18.37

Abbreviation: MMSE, Mini-Mental State Examination.

**Table 2:** Comparison of the Frequency of Behavioral Psychological Symptoms of Dementia across four groups according to the Mini-Mental State Examination scores.

BPSD symptoms	N=28 MMSE ≥24	N=25 MMSE: 20–23	N=27 MMSE: 14–19	N=18 MMSE: 0–13	P-overall
Delusions	2 (7.14)	4 (16)	6 (22.2)	7 (38.9)	0.067*
Hallucinations	2 (7.14)	4 (16)	5 (18.5)	6 (33.3)	0.165+
Agitation	9 (32.1)	7 (28)	13 (48.1)	10 (55.6)	0.188*
Depression	13 (46.4)	11 (44.0)	12 (44.4)	10 (55.6)	0.875*
Anxiety	26 (92.9)	15 (60.0)	14 (51.9)	5 (27.8)	<0.001*
Euphoria	0 (0.00)	3 (12.0)	1 (3.70)	5 (27.8)	**0.006+**
Apathy	2 (7.14)	5 (20.0)	7 (25.9)	9 (50.0)	**0.010+**
Disinhibition	7 (25.0)	7 (28.0)	8 (29.6)	6 (33.3)	0.942*
Irritability	12 (42.9)	10 (40.0)	12 (44.4)	10 (55.6)	0.773*
Aberrant motor behavior	0 (0.00)	3 (12.0)	8 (29.6)	15 (83.3)	**<0.001+**
Nighttime behavioral disturbances	21 (75.0)	13 (52.0)	14 (51.9)	15 (83.3)	0.052*
Appetite/Eating abnormality	7 (25.0)	5 (20.0)	8 (29.6)	12 (66.7)	0.007*

Abbreviations: BPSD, Behavioral Psychological Symptoms of Dementia; MMSE, Mini-Mental State Examination.

Note: *χ^2^ test; +Fisher’s exact test.

**Figure 1:**
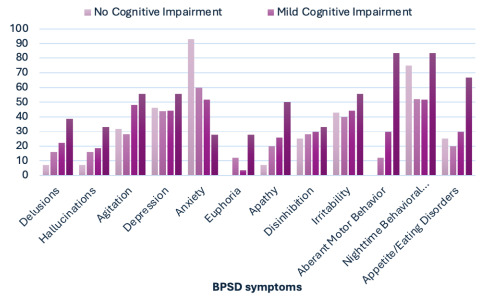
The distribution of behavior symptoms across the four groups classified based on the Mini-Mental State Examination.

 The most frequently reported symptoms were night-time behavioral disturbances, anxiety, depression, and irritability (Figure 1). The least frequently observed symptoms were euphoria, hallucinations, and delusions. Anxiety symptoms were most prevalent in the group with MMSE≥24, with a gradual decline observed in groups with lower MMSE scores, reaching the lowest prevalence in the group with MMSE<13. In contrast, motor disturbances, eating disorders, and nighttime behavioral disturbances were most frequently observed in the group with MMSE<13. These four symptoms demonstrated statistically significant differences (p<0.05) (Table 1). 

 Table 3 shows the median composite scores of the NPI-Q domains, indicating that the group with MMSE<13 had the highest total NPI-Q severity and distress scores. Differences in total NPI-Q severity and distress scores were statistically significant among the four groups stratified by MMSE score, as determined by Kruskal-Wallis analysis (p<0.05) 

**Table 3:** Comparison of Median Total Neuropsychiatric Inventory Questionnaire and Median total distress Neuropsychiatric Inventory score across four patient groups

All groups	N=28 MMSE ≥24	N=25 MMSE: 20–23	N=27 MMSE: 14–19	N=18 MMSE: 0–13	P-overall
Median + IQR total NPI-Q score	5.00 [4.00–9.25]	7.00 [5.00–8.00]	7.00 [5.50–9.00]	11.0 [9.00–16.0]	**0.001**
Median + IQR total NPI-Q distress score	2.50 [1.00–3.25]	4.00 [2.00–5.00]	4.00 [3.00–6.00]	7.00 [5.00–8.75]	**<0.001**

Abbreviation: MMSE, Mini-Mental State Examination.

Note: Kruskall Wallis test: P=0.001.

### The relationship between the severity of cognitive impairment and the manifestations of behavioral and psychological disorders

**Figure 2:**
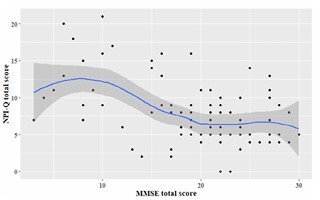
Correlation between the Mini-Mental State Examination score and Total score.

 Correlational analysis revealed a moderate inverse correlation between MMSE scores and total NPI-Q scores (Spearman’s rho=-0.38, p<0.001). A clear correlation was observed at an MMSE cut-off score of 20 with the total NPI-Q score. As MMSE scores decreased from 20 to 10, total NPI-Q scores increased from 8 to 13, indicating that in patients with moderate to severe cognitive impairment, behavioral and psychological symptoms progressively worsened. In the MMSE range of 20<MMSE≤30, the correlation between MMSE and NPI-Q scores was minimal, suggesting that cognitive function remained relatively preserved and that MMSE may not adequately differentiate levels of cognitive impairment in this range. At MMSE scores <10, patients exhibited severe cognitive decline accompanied by high NPI-Q scores (Figure 2). However, the severity of cognitive impairment at this stage requires further evaluation using additional cognitive scales beyond MMSE. 

## DISCUSSION

 This prospective study from the Southern Vietnam Dementia Research Center reveals that BPSD is common among patients with memory complaints, regardless of the severity of cognitive impairment. Findings indicate that lower MMSE scores are associated with more severe BPSD, with variations observed across different cognitive impairment groups. The prevalence of BPSD in this study (95.9%) was higher than in other global reports, likely due to the inclusion of patients aged 40 and above. BPSD is frequently reported as an early indicator of impending cognitive decline. Recent evidence suggests that subtle BPSD during the preclinical stage may signal the progression to cognitive impairment, making them valuable for early detection. In this study, 100% of patients with MMSE scores ≥24, classified as having subjective cognitive decline (SCD), exhibited BPSD. Anxiety was the most prevalent symptom, followed by nighttime behaviors, depression, and agitation. Symptoms such as apathy, hallucinations, and delusions were observed at a low rate (7.14%), and there were no signs of motor behavior abnormalities or euphoria. These findings are consistent with those of Sannemann et al., who observed that patients in the SCD group had significantly higher depression and anxiety scores compared to a healthy control group^11^. Differences in behavioral manifestations across stages are also noteworthy. A study by Adrienne L. Atayde et al. demonstrated a correlation between nighttime behaviors and more rapid cognitive deterioration^12^. 

 SCD refers to subjective cognitive complaints without objective evidence of impairment. It involves self-reported changes, often noticed by the individual, and is typically considered a potential early stage in the continuum toward MCI and AD^13,14^. Mild Behavioral Impairment (MBI), by contrast, focuses on behavioral and psychological changes that are observable by others and may precede or accompany cognitive decline. MBI involves changes in behavior and mood, often noticed by others, though the individual may also be aware. MBI is a risk marker for neurodegenerative disease and can be used to identify individuals at higher risk for developing dementia^15^. These findings are consistent with other studies showing that SCD patients present significantly higher anxiety and depression scores compared to healthy individuals, supporting the use of MBI as a diagnostic tool for identifying those at elevated risk for dementia. Understanding both SCD and MBI may aid early detection and intervention in neurodegenerative diseases^16^. 

 Previous studies, such as those by Yong Tae Kwak, have observed that anxiety tends to decrease as dementia progresses^17^. The current results show that anxiety was most common in patients with MMSE scores >24 (SCD) at 92.2% declining to 27.8% in those with MMSE scores <13 (Table 2). This suggests that more severe cognitive impairment is associated with reduced expression of anxiety, possibly due to difficulty distinguishing anxiety from depression and the overall progression of dementia. Depression was consistently observed across MMSE subgroups, appearing in 46 to 56% of patients, with no significant differences between groups. This supports Enache’s findings that depression is prevalent across all dementia types and stages, including MCI^18^. Psychotic symptoms such as delusions, hallucinations, eating disturbances, withdrawal, and motor disturbances were more frequent in patients with MMSE scores <13, indicating late-stage cognitive decline (Table 2). Such symptoms are common in AD and serve as predictors of accelerated cognitive and functional deterioration. In particular, the presence of hallucinations has been strongly associated with increased risk of institutionalization and higher mortality rates^19^. These results are aligned with findings by Wise et al.^20^, who observed that apathy, agitation, hallucinations, and delusions were rare prior to diagnosis in a U.S. cohort transitioning from MCI to dementia. However, severe BPSD can complicate diagnosis, especially in non-specialized centers. Patients with dementia who present with severe behavioral and psychological symptoms, particularly delusions or psychosis, may be misdiagnosed and managed as having primary psychiatric disorders. Such misdiagnosis may lead to inappropriate treatment approaches that overlooking the underlying neurodegenerative condition^21^. In many LMICs, dementia symptoms are frequently mistaken for typical signs of aging and are not perceived as requiring medical attention. This misconception, compounded by low awareness and stigma, contributes to delay help-seeking and treatment. 

 A moderate correlation was found between MMSE scores and BPSD severity. Among patients with moderate to severe cognitive impairment (MMSE 10–20), total NPI-Q scores increased from approximately 8 to 13 points (Figure 2). This is clinically relevant, as worsening cognitive function is correlated with increased BPSD severity, which often prompts caregiver concern and medical consultation. Family members are typically able to detect neurological or psychiatric issues at this stage, encouraging them to seek medical evaluation. The study by Solaphat Hemrungrojn et al.^8^, conducted in the Thai population, also demonstrated a correlation between cognitive function and behavioral symptoms. Additional studies by Manny have also confirmed the association between AD and behavioral symptoms^9^. 

 The present study found that as cognitive impairment worsens, both BPSD severity and caregiver distress increase (Table 3). The total NPI-Q distress scores may vary between countries due to cultural and societal factors, such as perceptions of aging and dementia, intergenerational living traditions, and caregiver responses to behavioral symptoms. However, based on clinical observation, the total NPI-Q score for neuropsychiatric symptoms may remain consistent across patients. Cultural and geographic factors, including living arrangements and societal attitudes toward older adults with dementia, may influence caregiver distress in response to BPSD. 

 This study is limited by its single-center design, relatively small sample size, and on the use of MMSE without adjustment for educational level. Additionally, caregiver perceptions of neuropsychiatric symptoms, such as distinguishing between anxiety and depression or between apathy and depression in advanced dementia, may pose challenges for accurate symptom assessment. Future research should include larger, multi-center samples and more comprehensive cognitive assessments. 

 In conclusion, the prevalence of behavioral and psychological symptoms in individuals presenting with memory complaints is notably high during initial dementia screening. A moderate negative correlation was observed between MMSE and NPI-Q scores. Neuropsychiatric symptoms may serve as early indicators of cognitive decline, particularly in individuals with SCD or mild cognitive impairment (MCI), highlighting their potential value in early screening strategies. 

 The combined use of MMSE and NPI-Q offers a simple, cost-effective approach that may help bridge diagnostic gaps in LMICs such as Vietnam, supporting earlier identification and timely intervention. 

## Data Availability

The data supporting the findings of this study is available from the corresponding author upon reasonable request.
